# Recurrent miscarriage and low-titer antiphospholipid antibodies

**DOI:** 10.1007/s10067-023-06843-x

**Published:** 2024-02-26

**Authors:** Jian Chen, Jing Yue, Yu Lu, Ting Li, Xue Li, Jian-Yu Zhang

**Affiliations:** Department of Rheumatology, Guangdong Provincial Key Laboratory of Major Obstetric Diseases, Guangdong Provincial Clinical Research Center for Obstetrics and Gynecology, The Third Affiliated Hospital of Guangzhou Medical University, Guangzhou, China

**Keywords:** Antiphospholipid, Antiphospholipid syndrome, Pregnancy, Prognosis

## Abstract

**Objective:**

To evaluate the clinical features and pregnancy outcomes in patients experiencing recurrent miscarriage (RM) with either low-titer or medium-high titer positivity of antiphospholipid antibodies (aPL).

**Methods:**

A retrospective review of medical records was conducted for patients with aPL positivity and recurrent miscarriage between 2018 and 2022. The clinical features, treatment strategies, outcomes were compared between the patients with low (*n* = 92) and medium (*n* = 32) titer of aPL.

**Results:**

A total of 118 patients, resulting in 124 obstetric episodes (pregnancies), with a mean age of 33. 15 ± 4.56 and 31.47 ± 4.41 years between the two groups. The low-titer group exhibited a higher frequency of anti-cardiolipin antibodies IgM (*P* < 0.001), whereas the medium-high titer group demonstrated a higher frequency of anti-β2-glycoprotein 1 antibodies IgG (*P* < 0.001) and IgM (*P* = 0.032). Moreover, the medium-high titer group displayed a significantly elevated erythrocyte sedimentation rate compared to the low-titer group (P < 0.05). In the low-titer group, 71 patients (77.2%) received appropriate treatment, resulting in 48 live births (67.6%) and 23 repeat abortions (32.4%). In the medium-high titer group, 29 patients (90.6%) received relevant treatment, leading to 23 live births (79.3%) and 6 repeat abortions (20.7%). No significant differences were observed in live births or maternal-fetal complications between the two groups (all *P* > 0.05).

**Conclusion:**

Noteworthy distinctions in laboratory parameters were identified between the low-titer and medium-high titer groups. However, when appropriately treated, the fetal-maternal outcomes were comparable in both groups. Timely intervention by clinicians is imperative to enhance pregnancy outcomes in patients experiencing recurrent miscarriage with low levels of aPL.

**Table:** 

**Key Points** • *This study challenges the conventional belief that only the higher antiphospholipid antibodies (aPL) titers directly correlated with worse pregnancy outcomes, which emphasized the importance of patients with low titer positive aPL-positive RM.* • *The results underscore the need for timely intervention in women with low titer aPL-positive RM, as it leads to favorable maternal–fetal outcomes.*

## Introduction

Recurrent miscarriage (RM) occurs in 0.4 to 2% of couples attempting conception, as determined by various criteria [1]. It is associated with anatomical, hormonal, and chromosomal abnormalities, as well as antiphospholipid syndrome (APS), a systemic autoimmune disease. APS is characterized by persistent positivity for antiphospholipid antibodies (aPLs), which lead to pathological pregnancy and thrombosis [2]. Obstetric APS (OAPS) is diagnosed when pathological pregnancy becomes the primary clinical feature [3]. APS accounts for 15% of miscarriages, with 50% of patients having an unknown cause [4]. The laboratory criteria for APS was based on the 2023 ACR/EULAR antiphospholipid syndrome classification criteria [5].

In certain cases of unexplained RM, low−titer aPL is observed, but it does not meet the APS criteria. A comparative analysis of pregnancy complications between patients with low and medium–high titers did not show any significant difference [6]. This finding suggests that a low titer of aPL could not adopted solely to exclude the diagnosis of OAPS. Other studies have shown that untreated patients with low−titer aPL, who do not meet the OAPS criteria, experience similar pregnancy outcomes to those who meet the criteria. However, treatment has shown promising results in improving pregnancy outcomes for patients with low−titer aPL [7, 8]. The role of positive lupus anticoagulant (LA) results in assessing the risk associated with low−titer aPL has also been implicated [9]. In 2013, a retrospective study included 139 pregnancies in antiphospholipid−positive women who were not fulfilling criteria for APS, and indicated that treatment may not provide additional benefits in terms of improving pregnancy outcomes for patients with low−titer aPL who do not meet the OAPS criteria [10]. But this study looking only at the effects of low−dose aspirin on pregnancy outcomes. Additionally, another study of 1640 patients published by Jaume Alijotas−Reig et al. showed the opposite findings, in which patients with OAPS and NC−OAPS women were put on preconceptional low−dose aspirin (LDA) plus prophylactic low molecular weight heparin (LMWH) from the first trimester [8].

Consequently, there is a need for a comprehensive evaluation of the clinical features and outcomes associated with low−titer aPL. This study aims to address this knowledge gap by systematically investigating the impact of low−titer aPL in RM patients and comparing pregnancy outcomes between patients with low and medium–high aPL levels. And it may support Jaume Alijotas−Reig’s findings in a Chinese population of patients [8].

## Materials and methods

### Design and patients

Clinical data of patients with RM who sought treatment at The Third Affiliated Hospital of Guangzhou Medical University between January 2018 and July 2022 were reviewed. Inclusion criteria required patients to have experienced two or more consecutive spontaneous abortions (fetal loss before 28 weeks of gestation) and tested positive for aPL before subsequent pregnancies. Patients were categorized into two groups based on their aPL titers: The low−titer group had aPL titers ≥20 GPL/MPL but <40 GPL/MPL, while the medium–high−titer group had aPL titers ≥40 GPL/MPL (with medium level titers as 40–79 GPL/MPL and high level as ≥80 GPL/MPL).

Exclusion criteria were as follows: (1) maternal and paternal chromosomal abnormalities; (2) fetal chromosomal abnormalities; (3) severe liver or kidney dysfunction, as well as severe cardiovascular and cerebrovascular diseases; and (4) confirmed diagnoses of other autoimmune diseases, malignant tumors, severe infections, or mental illness. The study was approved by the ethnic committee of the Third Affiliated Hospital of Guangzhou Medical University and performed according to the principle of the Declaration of Helsinki. Oral consent for this study was given by all patients.

### General information and clinical data

A comprehensive compilation of demographic and clinical data was extracted using a standardized digital form. The recorded variables encompassed age, body mass index (BMI), comorbidities, medication utilization, and the outcomes of subsequent pregnancies during the follow−up period. Additionally, a meticulous assessment of laboratory data was conducted, including the profile of antiphospholipid antibodies (aPLs), namely IgG/IgM anticardiolipin (aCL), IgG/IgM anti−β2−glycoprotein−1 (aβ2GP1), and lupus anticoagulant (LA). Furthermore, various other laboratory parameters, such as antinuclear antibody (ANA), anti−SSA/SSB antibody, anti−thyroglobulin (TGAb), thyroid peroxidase antibody (TPOAb), complement C3/C4 levels, immunoglobulin levels (IgG, IgA, IgM), C−reactive protein (CRP), and erythrocyte sedimentation rate (ESR), were scrupulously documented.

### Statistical analysis

The Shapiro–Wilk normality test assessed the distribution of continuous variables, while the Levene chi−square test examined the homogeneity of variance across groups. The Mann–Whitney *U*−test was applied to compare variables with skewed distributions. The results for non−normally distributed variables or those with unequal variances were reported as median (interquartile range (IQR) [median (P25, P75)]). Group comparisons were conducted using the Kruskal–Wallis test. For unordered categorical data, percentage analysis, cardinality tests, or Fisher’s exact probability tests was employed. When analyzing ordered categorical data, the Wilcoxon rank−sum test was used. All statistical analyses were performed using SPSS software (version 26.0, IBM, USA). Statistical significance was set at *P* < 0.05, indicating the presence of statistically significant differences.

### Patient and public involvement statement

Neither the patients nor the public actively participated in the design, execution, reporting, or dissemination plans of this research. The study solely relied on existing clinical and laboratory data, ensuring the preservation of patient privacy and confidentiality throughout the entire research process.

## Results

### General clinical information

A total of 118 women with 124 obstetric episodes (pregnancies) were included in the study (Table 1). The patients were categorized into two groups based on their pre-pregnancy antiphospholipid antibody (aPL) levels: the low-titer group (*n* = 92) and the medium-high-titer group (*n* = 32, with 17 cases of medium titer and 15 cases of high titer). There were no significant differences observed in terms of age, BMI, assisted reproduction treatment rate, and number of previous miscarriages between the two groups (*P* > 0.05). Hypertension and diabetes were not observed as comorbidities in either group. Among the participants in the low-titer group, five cases (5.4%) had hyperthyroidism, and three cases (3.3%) had hypothyroidism. In the medium-high-titer group, there were two cases (6.3%) of hypothyroidism, while no instances of combined hyperthyroidism were reported. The difference in comorbidity between the two groups was not statistically significant (*P* > 0.05), and the thyroid function remained within the normal range throughout the treatment period.

**Table 1:** Demographic characteristics of the included patients

	Low titers	Medium–high titers	*P*
No. of patients	92	32	
Age (years)	33.15 ± 4.56	31.47 ± 4.41	0.072
BMI (kg/cm^2^)	21.48 ± 3.04	21.00 ± 2.46	0.509
Hyperthyroidism	5 (5.4)	0 (0)	0.410
Hypothyroidism	3 (3.3)	2 (6.3)	0.827
Previous pregnancy losses	2 (2.3)	2 (2.2)	0.126
Conception method			0.884
Natural conception, *n* (%)	73 (79.3)	25 (78.1)	
Assisted reproduction, *n* (%)	19 (20.7)	7 (21.9)	

*BMI* body mass index

### aPL spectrum and relevant laboratory features

Significant variations were observed in the different isotypes between the two groups. ACL−IgM exhibited a higher prevalence in the low−titer group compared to the medium–high−titer group (81.5% vs. 37.5%, *P* < 0.001), and it constituted the predominant antibody isotype in the low−titer group. Conversely, aβ2GP1−IgG demonstrated the highest positivity rate in the medium–high−titer aPL group (40.6%) and the lowest in the low−titer aPL group (3.3%), with a notable difference (*P* < 0.001). The medium–high−titer group displayed a significantly higher positive rate of aβ2GP1−IgM compared to the low−titer group (31.3% vs. 14.1%, *P* = 0.032), while no significant difference was observed between the two groups for ACL−IgG. None of the patients in the low−titer group exhibited triple positivity, whereas two patients (6.3%) in the medium–high−titer group demonstrated triple positivity. The proportion of single positive antibodies was higher in the low−titer group (90.2%), while the medium–high−titer group had a higher proportion of double positive antibodies (12.5%) and LA positivity (7.4%), although these differences were not statistically significant (Table 2).

**Table 2:** The aPL and other laboratory profiles of the patients

	Low titers (*n* = 92)	Medium–high titers (*n* = 32)	*P*
aβ2GP1-IgG positive	3 (3.3%)	13 (40.6%)	< 0.001
aβ2GP1-IgM positive	13 (14.1%)	10 (31.3%)	0.032
ACL-IgG positive	9 (9.8%)	7 (21.9%)	0.147
ACL-IgM positive	75 (81.5%)	12 (37.5%)	< 0.001
LA positive	3 (3.8%)	2 (7.4%)	0.812
Single positive	83 (90.2%)	26 (81.3%)	0.305
Double positive	9 (9.8%)	4 (12.5%)	0.923
Triple positive	0 (0%)	2 (6.3%)	0.109
ANA positive	12/76 (15.8%)	7/28 (25%)	0.281
Anti-SSA antibody positive	7/72 (9.7%)	3/27 (11.1%)	1.000
Anti-SSB antibody positive	0 (0%)	1/27 (3.7%)	0.614
TGAb positive	5/75 (6.7)	1/27 (3.7%)	0.933
TPOAb positive	9/75 (12)	3/27 (11.1%)	1.000
IgG (g/L)	12.28 ± 2.14	13.24 ± 2.40	0.707
IgA (g/L)	2.28 (1.91, 2.75)	2.90 (1.87, 3.31)	0.445
IgM (g/L)	2.06 ± 0.86	1.93 ± 0.79	0.072
C3 (g/L)	1.17 (1.10, 1.31)	1.33 (1.18, 1.50)	0.124
C4 (g/L)	0.22 (0.18, 0.29)	0.26 (0.15, 0.31)	0.566
CRP (mg/L)	0.75 (0.33, 2.45)	3.26 (2.47, 5.11)	0.144
ESR (mm/h)	11 (5, 16)	19.5 (11.77, 25)	0.006

*aβ2GP1* anti-β2-glycoprotein 1 antibodies, *aCL* anticardiolipin antibodies, *LA* lupus anticoagulants, *ANA* antinuclear antibody, *TGAb* thyroglobulin autoantibodies, *TPOAb* anti-thyroperoxidase antibodies, *ESR* erythrocyte sedimentation rate, *CRP* C-reactive protein

Furthermore, the medium–high−titer group exhibited significantly higher levels of ESR compared to the low−titer group (*P* = 0.006). There were no differences in the rates of autoantibody positivity for ANA, SSA, SSB, TGAb, and TPOAb between the two groups.

### Treatment strategies

There were no statistically significant differences observed in the treatment received between the low−titer group and the medium–high−titer aPL group (*P* > 0.05).

In the low−titer group, 71 out of 92 patients (77.2%) underwent treatment, while the remaining 21 patients (22.8%) did not receive any treatment (Table 3). The majority of patients (47.8%) received a comprehensive treatment regimen consisting of LDA, LMWH, glucocorticoids (GCs), and hydroxychloroquine (HCQ). Other therapeutic options is as follows: One patient (1.0%) received low−dose aspirin (LDA) only, 3 patients (3.3%) received low molecular weight heparin (LMWH) only, 11 patients (12%) received a combination of LDA and LMWH, and 4 patients (4.4%) received a combination of low−dose GCs along with LDA and LMWH. Furthermore, eight patients (8.7%) received LDA, LMWH, and HCQ.

**Table 3:** Treatment options for patients with low and medium–high titers of aPL

	Low titers (*n* = 92)	Medium–high titers (*n* = 32)	*P*
Without treatment	21 (22.8%)	3 (9.4%)	0.097
LDA alone	1 (1.0%)	0 (0%)	0.554
LMWH alone	3 (3.3%)	3 (9.4%)	0.165
LDA + LMWH	11 (12.0%)	2 (6.2%)	0.364
LDA + LMWH + GCs	4 (4.4%)	1 (3.1%)	0.762
LDA + LMWH + HCQ	8 (8.7%)	4 (12.5%)	0.531
LDA + LMWH + GCs + HCQ	44 (47.8%)	19 (59.4%)	0.260

*LDA* low-dose aspirin, *LMWH* low molecular weight heparin, *HCQ* hydroxychloroquine, *GCs* glucocorticoids

In the medium-high-titer aPL group, 29 out of 32 patients (90.6%) received treatment, while 3 patients (9.4%) did not undergo any treatment. The majority of patients (59.4%) in this group received a comprehensive treatment regimen comprising LDA, LMWH, GCs, and HCQ. Other therapeutic options is as follows: Three patients (9.4%) received LMWH only, two patients (6.2%) received a combination of LDA and LMWH, one patient (3. 1%) received a combination of LDA, LMWH, and GCs, and four patients (12.5%) received a combination of LDA, LMWH, and HCQ (Table 3).

### Pregnancy outcome

Among the 71 patients in the low−titer aPL group who received treatment, 48 patients (67.6%) achieved successful live births, while 23 patients (32.4%) experienced recurrent abortions, primarily consisting of 21 cases (91.3%) classified as early fetal losses. In the medium–high−titer aPL group, 23 out of 29 patients (79.3%) had successful live births, while 6 patients (20.7%) faced recurrent miscarriages. Statistical analysis revealed no significant difference in the rates of miscarriage and maternal–fetal complications between the two groups. However, it is noteworthy that the rates of miscarriage were higher in both the low−titer (66.7%) and medium–high−titer (33.7%) groups of untreated aPL−positive patients compared to the treated group (Table 4).

**Table 4:** Pregnancy outcomes in low- and medium–high-titer aPL-positive patients

	With treatment (*n* = 100)			Without treatment (*n* = 24)		
	Low titers (*n* = 71)	Medium–high titers (*n* = 29)	*P*	Low titers (*n* = 21)	Medium–high titers (*n* = 3)	*P*
Live birth	48 (67.6%)	23 (79.3%)	0.242	7 (33.3%)	2 (66.7%)	0.633
Miscarriage	23 (32.4%)	6 (20.7%)	0.242	14 (66.7%)	1 (33.7%)	0.633
Early miscarriage	21 (91.3%)	5 (83.3%)	1.000	14 (100%)	1 (100%)	-
Late miscarriage	2 (8.7%)	1 (16.7%)	1.000	0 (0%)	0 (0%)	-
Weight of newborn (g)	3270 (3200, 3500)	3050 (2785, 3240)	0.889	3270 (3200, 3500)	2065 ± 487.9	0.143
Maternal–fetal complications						
Pregnancy hypertension	1 (2.1%)	3 (13%)	0.185	0 (0%)	0 (0%)	-
Pre-eclampsia	1 (2.1%)	2 (8.7%)	0.506	0 (0%)	0 (0%)	-
Gestational diabetes mellitus	5 (10.4%)	3 (13%)	1.000	1 (14.3%)	1 (50%)	0.915
Postpartum hemorrhage	0 (0%)	3 (13%)	0.054	1 (14.3%)	0 (0%)	-
Fetal growth restriction	1 (2.1%)	0 (0%)	1.000	0 (0%)	0 (0%)	-
Low birth weight infant	4 (8.3%)	1 (4.3%)	0.906	1 (14.3%)	1 (50%)	0.915
Premature rupture of membranes	5 (10.4%)	7 (30.4%)	0.077	1 (14.3%)	1 (50%)	0.915
Preterm delivery	1 (2.1%)	2 (8.7%)	0.506	1 (14.3%)	2 (100%)	0.156
Fetal distress	9 (18.8%)	2 (8.7%)	0.456	0 (0%)	0 (0%)	-

## Discussion

This study comprehensively evaluated the clinical feature and pregnancy outcomes among patients with RM and aPL positive in China. The results demonstrated that appropriately treated patients in both low-titer and medium-high-titer aPL positivity achieved higher live birth rates. These findings highlight the necessity of actively intervening for patients with RM, even with low-titer aPL positivity, potentially enhancing pregnancy outcomes.

Spontaneous abortion, a common complication affecting around 20% of pregnant women, recurs in approximately 30% of women following two consecutive miscarriages [11]. Obstetric antiphospholipid syndrome (OAPS) is recognized as a prevalent acquired risk factor for recurrent miscarriages [12]. The prevalence of antiphospholipid (aPL) positivity in women with recurrent miscarriages is approximately three times higher than in women with normal pregnancies [13]. Among OAPS patients, recurrent miscarriage represents the most frequent adverse pregnancy outcome, accounting for 38.6% of cases [14]. While consistently positive medium–high titers of aPL serve as established laboratory criteria for diagnosing OAPS, cases with low aPL titers or a single positive test are classified as non−criteria OAPS [4]. In contrast to the “second strike” theory of APS thrombosis, OAPS patients exhibit elevated β2GP1 levels in placental endothelial cells, syncytial trophoblasts, and extravillous trophoblasts, resulting in aPL binding without a “second strike” and leading to various mechanisms of adverse pregnancy outcomes, even at low aPL titers [15]. Consequently, the diagnostic threshold for OAPS during pregnancy is likely to be lower than the classification criteria [16, 17].

A retrospective cohort study demonstrated that over 50% of women exhibiting clinical features of OAPS, but without thrombosis, display low titers of positive anticardiolipin (aCL) and/or anti−β2GP1 antibodies [18]. Ofer−Shiber et al. [6] conducted a study investigating the incidence of thrombosis and obstetric complications in patients with low and medium–high titers of aPL and found no significant difference between these groups. Retrospective and prospective studies indicated that low aPL titers (defined as the 95th and 99th percentiles) are clinically relevant in women with pure OAPS [1, 7, 18, 19]. Our study revealed significant improvements in the live−birth rate after treatment, with no statistical differences observed in the miscarriage rate, neonatal weight, or maternal–fetal complication rate between the two groups, which was consistent with these studies.

In the 2020 study based on the European OAPS registry, the laboratory characteristics and maternal–fetal outcomes of 1000 OAPS patients and 640 patients with non−criteria OAPS (including 175 patients with low−titer aPL/intermittent positive aPL) were compared [8]. Despite significant differences in aPL profiles between the two groups, similar positive maternal–fetal outcomes were observed following treatment. The authors concluded that treatment during pregnancy was highly beneficial for these patients. The findings of the present study also support this conclusion. More recently, the EUREKA algorithm assessed the risk of aPL−related pregnancy morbidity based on aPL titers and antibody profiles, revealing a significant impact of aPL on pregnancy morbidity even at low titers and when meeting the clinical classification criteria for OAPS. Notably, patients with low titers derived even greater benefit from treatment compared to those with criteria aPL titers [20].

Rai et al. proposed that untreated recurrent miscarriage (RM) patients with consistently low levels of anticardiolipin (aCL) antibodies (<99th percentile) experienced fetal loss rates exceeding 90% [2]. Furthermore, a meta−analysis indicated that both low and medium–high titers of aCL were associated with early (OR = 3.56) and late recurrent pregnancy loss (OR = 3.57) [21]. Consistent with these findings, our study revealed that ACL−IgM antibodies were the predominant isotype in the low−titer aPL group (81.5%). Compared with the low−titer group, the medium–high−titer group demonstrated significantly higher positivity for aβ2GP1−IgG (40.6% vs. 3.3%, *P* 0.001) and aβ2GP1−IgM (31.3% vs. 14. 1%, *P* = 0.032). However, there was no significant difference in pregnancy complications between the two groups, which precisely indicates that women with low−titer aPL−positive RM should receive the same attention as those with medium–high titers.

The EULAR 2019 and ACR 2020 guidelines recommend the use of LDA and/or LMWH for OAPS patients who meet the diagnostic criteria, respectively [22, 23]. In contrast, for non−criteria OAPS patients with aPL titers below diagnostic criteria, the EULAR guidelines recommend LDA and/or LMWH, while the ACR guidelines only recommend LDA. The lack of consensus on treatment may lead clinicians to empirically treat patients. A recent systematic review of placental histopathology in aPL−positive women identified common features associated with aPL, including placental infarction, impaired spiral artery remodeling, decidua inflammation, increased syncytial cell nodules, reduced vascular smooth muscle cell membranes, and complement split−product deposition [24]. These findings suggest the involvement of angiogenic and inflammatory factors in the pathology of the disease. β2GP1, targeted by aPL, is expressed on trophoblast cell surfaces and activates the Toll−like receptor 4/MyD88 pathway, promoting the secretion of pro−inflammatory cytokines and chemokines [25]. Heparin not only exhibits an antithrombotic effect but also inhibits complement activation and the binding of aPL to trophoblast cells, thus exerting an inhibitory effect on inflammation in aPL−associated pathological pregnancies.

In clinical practice, despite the combination of LDA and LMWH, a significant proportion of patients (20–30%) continue to experience RM [26]. However, the addition of anti−inflammatory treatment (prednisone + hydroxychloroquine) to anticoagulation (LDA + LMWH) demonstrates a notable reduction in the incidence of recurrent miscarriage compared to anticoagulation alone (LMWH + LDA) (22.70% vs. 11.11%) [27]. APLs have the potential to induce an inflammatory response in trophoblast cells, resulting in the release of tumor necrosis factor and activation of neutrophils. In an experimental mouse model of aPL−induced fetal loss, the inhibition of tumor necrosis factor production effectively prevents placental damage [28]. The robust inflammatory response may also interfere with the early stages of embryo implantation, providing an explanation for the potential involvement of aPL in early abortion [29]. Animal models investigating aPL−mediated fetal loss have exhibited the infiltration of inflammatory cells in the placenta, thus suggesting the contribution of inflammation to fetal loss [30]. The early initiation of anticoagulation and anti−inflammatory therapy can effectively suppress the autoimmune inflammatory response at the maternal–fetal interface, leading to improved trophoblastic and placental function and a reduction in adverse pregnancy outcomes [9].

HCQ is extensively utilized in patients with autoimmune disorders due to its anti−inflammatory and immunomodulatory properties. In vitro studies have demonstrated that HCQ’s immunomodulatory effects may confer beneficial outcomes on pregnancy in individuals positive for aPL [31]. Furthermore, HCQ hinders the release of inflammatory cytokines, interferes with Toll−like receptor−mediated innate immune responses, impedes the antigen presentation process, and inhibits platelet aggregation and activation [32]. HCQ also inhibits complement activation, ameliorates placental dysfunction, and fosters normal fetal brain development [33]. These findings substantiate the potential of HCQ as a promising therapeutic approach to enhance pregnancy outcomes among women experiencing recurrent miscarriages and testing positive for aPL.

The present study has some limitations. Firstly, its retrospective design introduces inherent biases and constraints in data collection and analysis. Secondly, the study had a limited sample size, which may restrict the generalizability of the results to a broader population. Thirdly, the study focused on a Chinese population and setting, limiting the applicability of the findings to other ethnicities. In addition, because the study was retrospective, there was significant variability in the treatment regimens used in both groups, which may lead to misleading results. Finally, as with any observational study, the presence of confounding factors cannot be entirely eliminated, and establishing causality remains inconclusive. Notwithstanding these limitations, the study findings contribute valuable insights to the field and underscore the necessity for future research with larger cohorts and prospective designs to corroborate and broaden the scope of the observed outcomes.

## Conclusion

In conclusion, the study findings indicate similar fetal-maternal outcomes in low and medium–high aPL-positive RM patients after treatment, despite variations in laboratory characteristics. Neglecting patients with low titer or intermittently positive aPL-positive RM, who do not meet APS classification criteria, increases the risk of recurrent miscarriage. The study confirms the positive influence of timely intervention on favorable maternal fetal outcomes in patients with low-titer aPL-positive RM and supports Jaume Alijotas-Reig’s findings on OAPS in a Chinese population of patients. Further well-designed prospective multicenter studies are needed to validate these findings.

## Data Availability

The data used or analyzed during the current study are available from the corresponding author on reasonable request.
